# Mapping socio-geographical disparities in the occurrence of teenage maternity in Colombia using multilevel analysis of individual heterogeneity and discriminatory accuracy (MAIHDA)

**DOI:** 10.1186/s12939-024-02123-5

**Published:** 2024-02-23

**Authors:** Hedda Mattsson, Johanna Gustafsson, Sergio Prada, Laura Jaramillo-Otoya, George Leckie, Juan Merlo, Merida Rodriguez-Lopez

**Affiliations:** 1 Unit for Social Epidemiology, Faculty of Medicine, Lund University, Malmö, Sweden; 2 Fundación Valle del Lili, Centro de Investigaciones Clínicas, Cali, Colombia; 3 Universidad Icesi, Centro PROESA, Cali, Colombia; 4 Faculty of Health Science, Pontificia Universidad Javeriana Cali, Cali, Colombia; 5 Centre for Multilevel Modelling, University of Bristol, Bristol, UK; 6 Faculty of Health Science, Universidad Icesi, Calle 18 No. 122 -135, Cali, Colombia

**Keywords:** Pregnancy in adolescence, Multilevel analysis, Intersectionality, Social inequity, Multilevel Analysis of Individual Heterogeneity and Discriminatory Accuracy (MAIHDA)

## Abstract

**Background:**

The prevalence of teenage pregnancy in Colombia is higher than the worldwide average. The identification of socio-geographical disparities might help to prioritize public health interventions.

**Aim:**

To describe variation in the probability of teenage maternity across geopolitical departments and socio-geographical intersectional strata in Colombia.

**Methods:**

A cross-sectional study based on live birth certificates in Colombia. Teenage maternity was defined as a woman giving birth aged 19 or younger. Multilevel analysis of individual heterogeneity and discriminatory accuracy (MAIHDA) was applied using multilevel Poisson and logistic regression. Two different approaches were used: (1) intersectional: using strata defined by the combination of health insurance, region, area of residency, and ethnicity as the second level (2) geographical: using geopolitical departments as the second level. Null, partial, and full models were obtained. General contextual effect (GCE) based on the variance partition coefficient (VPC) was considered as the measure of disparity. Proportional change in variance (PCV) was used to identify the contribution of each variable to the between-strata variation and to identify whether this variation, if any, was due to additive or interaction effects. Residuals were used to identify strata with potential higher-order interactions.

**Results:**

The prevalence of teenage mothers in Colombia was 18.30% (95% CI 18.20–18.40). The highest prevalence was observed in Vichada, 25.65% (95% CI: 23.71–27.78), and in the stratum containing mothers with Subsidized/Unaffiliated healthcare insurance, Mestizo, Rural area in the Caribbean region, 29.08% (95% CI 28.55–29.61). The VPC from the null model was 1.70% and 9.16% using the geographical and socio-geographical intersectional approaches, respectively. The higher PCV for the intersectional model was attributed to health insurance. Positive and negative interactions of effects were observed.

**Conclusion:**

Disparities were observed between intersectional socio-geographical strata but not between geo-political departments. Our results indicate that if resources for prevention are limited, using an intersectional socio-geographical approach would be more effective than focusing on geopolitical departments especially when focusing resources on those groups which show the highest prevalence. MAIHDA could potentially be applied to many other health outcomes where resource decisions must be made.

**Supplementary Information:**

The online version contains supplementary material available at 10.1186/s12939-024-02123-5.

## Introduction

Teenage pregnancy is usually defined as pregnancy occurring in a woman under the age of 20 [1, 2]. This population has an increased risk of complications such as eclampsia, infections, and obstetric fistula during childbirth [3], as well as adverse neonatal outcomes including premature birth [2]. In addition, teenage pregnancies have been associated with an increased risk of maternal and newborn mortality [1, 4]. Adolescent maternity has been associated with a higher risk of withdrawing from high school [5] and lower employment opportunities, predisposing to gender inequity across the life course. In addition, a multigenerational effect of this problem has been also documented, as children from adolescent mothers tend to have children who have behavioral problems and reduced educational attainment, perpetuating the cycle of poverty [6].

There is also a long-term financial cost to teenage maternity in both society and the state. At a societal level, there is a loss of human capital in the labor market, which causes reduced participation in the workforce for the mother or leads to employment in low-paid jobs [7]. The opportunity cost falls proportionally more on governments in countries with high rates of taxation and where the public health system has a wider coverage [8]. In Colombia, 97% of the opportunity cost associated with teenage maternity has been calculated to fall on individual mothers, and the relative contribution to labour income and education to the opportunity cost is 49% and 30%,respectively [8].

Even though there has been a downward trend in the prevalence of adolescent pregnancies over time [9], it is still considered a public health issue for all countries, especially those in developing regions [1]. Indeed, 45 per 1000 teenagers experience pregnancy in low to middle income countries worldwide compared to 10 per 1000 in high income countries [10]. Latin America and the Caribbean together have the second highest prevalence of teenage pregnancies of all regions in the world after sub-Saharan Africa. In 2022, the rates were 52.1 and 99.4 births per 1000 women, respectively [11]. In Colombia, the estimated prevalence of teenage pregnancy in 2015 was 17.4% [12], and the fertility rate in 2021 rose to 52.78 live births per 1000 women aged 15–19 years [13] In addition, adolescent birth rate is a reproductive health indicator included in Gender inequity index, along with maternal mortality. According to the United Nation Development Program in 2021, Colombia ranked 102 globally [14] with a higher index than those of other Latin American countries, including Chile, Uruguay, Costa Rica, Mexico, and Argentina, suggesting greater gender inequities [14]. Therefore, efforts are needed to reduce adolescent maternity in Colombia.

Specific sociodemographic determinants of health have been associated with teenage pregnancy [1, 15, 16]. In Colombia, a lack of health insurance, low education levels, and living in rural areas have all been associated with an increase in teenage pregnancy prevalence [17, 18]. However, the effect of social determinants has mainly been explored using one social dimension at a time or a limited number of dimensions together “adjusted for each other” using univariate or multivariate analysis, respectively [19]. Both approaches ignore the interlocking nature of the different dimensions. Similarly, conventional studies typically assume no interactive effects [20, 21] and, if such interactions are evaluated, they are usually limited to two variables at a time and using a fixed reference category [20], which neglects the existence of higher-way interactions of effects between three or more interlocking social dimensions. Intersectionality theory posits that social disparities emerge along multiple, non-independent, and possibly interacting categories. It proposes that social determinates of health, including gender, ethnicity, and socioeconomic position, should be understood as interwoven. These intersectional strata condition the distribution of resources and power in a society and thereby shape individual experiences and outcomes [22, 23].

Analyzing one social determinant at a time could lead to an unnecessary and groundless stigmatization of many individuals belonging to groups categorized as “high risk”, and also result in many cases being missed from those in supposed “low risk” groups. This situation may lead to ineffective public health interventions if decisions are only based on average risk differences, a phenomenon that has been referred to as the ‘tyranny of averages’ [24]. For instance, teenage pregnancies may be on average more frequent among girls from rural areas and low socioeconomic groups [17, 25], but many of those girls do not become pregnant at that age. In addition, in absolute figures, the number of teenage pregnancies could be higher in the groups of girls from urban areas and high socioeconomic positions if the underlying population sizes of these groups are larger. Therefore between-group and within-group heterogeneity need to be measured and considered simultaneously when interpreting measures of association in public health practice [26, 27]. Most intersectional analyses, including those related to teenage pregnancies, have used qualitative approaches [25, 28, 29]. More recently, an analytical approach has been developed to quantify the heterogeneity across social position [23, 28, 30, 31] and risk factors [32, 33] in health studies. To the best of our knowledge, an intersectional quantitative approach to map the distribution of teenage maternity remains little studied and underexplored.

In this study, we used a state-of-the-art analytical approach based on a multilevel analysis of individual heterogeneity and discriminatory accuracy (MAIHDA) [21, 34–36] to illustrate an analytical strategy that allows for the exploration of inequalities in the risk of teenage maternity in Colombia. We used information from live birth certificates, therefore, we referred to the outcome variable as teenage maternity instead of teenage pregnancy. We specifically discerned which of the following two approaches 1) socio-geographical and intersectional, where individuals (level 1) are nested within intersectional strata (level 2), or 2) merely a geographical multilevel analysis, where individuals (level 1) are nested within geopolitical departments (level 2), would better discriminate teenage mothers from adult mothers. By using MAIHDA, differences between Colombia’s geopolitical departments and intersectional strata are measured as the percentage of the total variance in the individual propensity for teenage maternity attributed to level 2 variables. Identifying the relevance of intersectional strata vs. departments in teenage pregnancy would guide stakeholders to determine whether healthcare interventions to decrease the risk of teenage maternity in Colombia should be focused, universal or follow a proportional universalism approach [28, 35–37] and so could inform decision-making on the allocation of scarce resources for public health or health service interventions and research.

## Methods

### Study design and participants

This is a cross-sectional study based on information on live birth certificates in Colombia from January 1st to December 31st 2020. We followed the STROBE (STrengthening the Reporting of OBservational studies in Epidemiology) Guidelines for reporting observational studies together with the LEVEL (Logical Explanations & Visualizations of Estimates in Linear mixed models) checklist for reporting multilevel results [38]. The information from the certificates is included in *the birth database*, a publicly available dataset, facilitated by the website of the National Administrative Department of Statistics (DANE): www.dane.gov.co  [39]. The information is regularly recorded by authorized health care workers such as physicians, nursing assistants, and health promoters, which are trained about the importance of the veracity and completeness of the data. The validity of birth certificate in Colombia has been substantially improved, and vital statistics data currently estimates that 97% of all births in Colombia are officially registered [40]. The data are fully anonymized and quality control processes are performed before they are made publicly available.

### Eligibility criteria

Our study population was limited to women who gave live birth to a single child due to the impossibility of identifying paired records in the database, and to those with information in maternal age. Hence, the number of live births corresponds to the number of pregnant women. Births with missing maternal age or socio-geographical variable data were excluded from the analysis, which represents 1.6% of births, meeting the eligibility criteria. Then a complete case analysis was performed.

### Assessment of variables

#### Outcome variable

We defined adolescence as the period between the ages of 10 and 19 years [1]. Therefore, we dichotomized mother’s age into teenagers aged 19 or below and adults aged 20 years and above.

#### Individual level variables

The mother’s affiliation to the General Health Social Security System includes four regimes of health insurance *(i)* The *contributive* including those who are employed, self-employed or pensioners. *(ii)* The *special or exception* including those who work within the armed forces, national police, public universities, national Colombian oil company, or as public teachers. *(iii)* The *subsidized regime* including those who cannot contribute to the general health social security system. First grade relatives and steady partners can also be affiliated as beneficiaries in these regimes, and, finally *(iv)* Unaffiliated including those below a poverty threshold measured by a means-test called SISBEN (System for the Identification of Potential Beneficiaries of Social Programs) [37]. Most Colombians belong to the contributive and subsidized regimes. This variable was dichotomized for the analysis by merging those categories with similar univariate risk of the outcome as follows: *(i)* Contributive/special or exception further called Contributive, and *(ii)* Subsidized/Unaffiliated.

Colombia is a multiethnic country. DANE officially recognizes five ethnic groups: *(i)* indigenous, *(ii)* Romani, *(iii)* Raizal, *(iv)* Palenquero, *(v)* Afro-Colombian. Indigenous usually represents the native South American population. Usually, those whites and mestizos are reported as "non-ethnic population” and we will further call “mestizos”. Due to the small sample of groups *ii* (*n* = 56) *iii* (*n* = 336) *and iv* (*n* = 63)*,* they were incorporated to group *v* and further classified as Afro-Colombian/Romani. Then, three categories of ethnicity were analyzed: *(i)* Indigenous, *(ii)* Afro-Colombian and *(iii)* Mestizos. Ethnicity is recorded in birth certificates by asking parents their child’s classification according to the culture, people or physical traits [39].

The degree of urbanization of the place where the women were residing was divided into: *(i)* urban area, and *(ii) rural* area which includes towns. There are six geographical regions in Colombia: (i) Andean, (ii), Amazon, (iii) Caribbean, (iv) Orinoco, (v) Pacific, and (vi) Island. Colombia is a multicultural country; regions are also culturally diverse with some degree of overlap between geographical and cultural regions. We decided to include the Island region into the Caribbean region since these share similarities, and because of the small sample size from the Island region. Hence, from now on, we will only mention five geographical regions.

#### Contextual variables

##### Intersectional

To study socioeconomic differences in the occurrence of teenage maternity we adopted an intersectional approach and used the dimensions explained above to define 60 intersectional contexts or strata resulting from combining the two categories of social security, the three categories of ethnicity, the two categories of urbanization, and the five geographical regions (i.e., 2 × 3 × 2 × 5 = 60).

##### Geographical

We also evaluate the contextual geographical effect of the political-administrative divisions existing in Colombia which include the capital district, Bogotá, and 32 departments. Each department has their own public health administration that leads, implement, and guides the formulation of public health policies, strategies, and plans. Therefore, geopolitical departments could also influence the prevalence of teenage maternity and so were included in our geographical analysis.

### Statistical methods

All statistical analyses were performed using Stata 14 (Stata Corp., College Station, TX, USA). The prevalence and associated 95% confidence interval (CI) of teenage maternity in the birth database was obtained for the country as a whole, for each department, and for each intersectional multi-categorical stratum to provide a detailed map of the observed absolute risk of teenage maternity. Thereafter, we conducted a conventional univariate Poisson regression of teenage maternity on each variable in turn to obtain the Prevalence Rate Ratio (PRR) for each factor. The category with the lower prevalence was considered as the reference category [41].

Then, we performed MAIHDA models distinguishing between two contexts: socio-geographical intersectional strata [21] and geographical strata [42]. Variables that define these contexts were included as level 2. Individuals were included as level 1. That is, the mother is considered nested within intersectional (socio-geographical) or department (geographical) strata. The geographical approach recreates the classical multilevel hierarchical structure. Likewise, Intersectional MAIHDA consider that women sharing the same strata with complex combination of social disadvantage (e.g., having the same ethnicity, urbanicity, region and health insurance) will tend to have similar risk of adolescent maternity, and therefore they will tend to have correlated outcomes within each stratum. A Stata dofile is provided as Supplementary material to replicate the analysis using the information provided in Tables 2 and 3.

#### Null models

In our first *empty or null intersectional model* we only included strata as a level 2 random effect in a MAIHDA Poisson regression. MAIHDA enables one to obtain precision-weighted, level 2-specific predictions using shrunken residuals. The model generates a predicted value that is unique for every stratum analyzed. Residuals are shrunken towards the overall mean based on the uncertainty in their estimate, which is an argued advantage of the approach [23, 43, 44]. The smaller the strata, the greater the shrinkage. Hence, the predicted teenage maternity prevalence obtained for small strata are shrunk more towards the overall average prevalence than those for large strata. This protects against the extreme results often associated with smaller strata.

The Variance Partition Coefficient (VPC) was calculated by dividing the stratum variance by the total variance. We obtained the VPC in two ways. First, in terms of the model implied observed outcome variance from multilevel Poisson regression (VPC_poisson_) [45]. Then, in terms of the model implied latent outcome variance as derived from the equivalent multilevel logistic regression (VPC_logit_) [45, 46]. The VPC from the null model quantifies the proportion of the total individual variance in the propensity of being a teenage mother that is at the strata level. In this way, this model measures the influence of intersectional or geographical strata on the outcome without specifying any specific characteristics of the strata. Therefore, this measurement is often referred to as the “general contextual effect” (GCE). A similar approach was used to identify the variation in the propensity of teenage maternity across geographical departments. The proportion of the total individual variation which lies within strata or departments, is therefore given by 1—VPC. Subscripts were used to identify results derived from Intersectional _(inter)_ and geographical _(geo)_ approaches.

##### Understanding the General contextual effect

The higher the VPC, or GCE, the larger the intersectional or geographical differences are [47]. Therefore, higher VPC values represent greater disparities in teenage maternity. Considering previous references [37, 47], the magnitude of the VPC_null-logit_ after multilevel logistic regression model was classified as absent if the VPC in the null model, was between: 0–1%, small between 1–5%, moderate between 5–10%, large between 10–20%, and very large > 20%. See elsewhere for an extended explanation of the GCE concept [32, 42, 48].

A different way of interpreting the GCE is by measuring the Area under the curve (AUC). This approach quantifies the accuracy of the strata/departments to distinguish teenage mothers from adult mothers [28, 29]. The AUC was based on the Poisson model prediction and can be classified as: absent or very small between 0.5–0.6, moderate between 0.6–0.7, large between 0.7–0.8, and very large between 0.8–1.0. This information of GCE helps to understand if potential interventions should be universal (i.e., absent or very small VPC and AUC), or targeted to specific contexts otherwise. In the latest case, proportional universalism approach can also be adopted [18, 23–25].

#### Partial models

If the VPC from the two null models were moderate and above, we included individual variables as fixed effects covariates. First, four partial or intermediate models were fitted to evaluate the contribution of each specific determinant to the between-stratum variance. Variables were entered in their dummy variable form. These intermediate models extend the null models by including one variable at a time. Thus, while the null models quantify the overall extent of stratum differences in teenage maternity prevalence, the intermediate models seek to explain these differences by estimating the relative role of each determinant used to create the strata. Determinants that define the strata are constant across individuals within each stratum. Therefore, the between-stratum variance summarizes the differences that remain between strata after taking into account the main effect of the included determinant. VPC_partial_ summarizes the degree of residual clustering, having adjusted for the included determinant. PCV_partial_ denotes the degree to which the between-stratum variance reduces as we move from the null to the intermediate models or the amount of between-stratum variance that is “explained” by including fixed effect of the covariate. A high PCV value indicates that the included variable has a substantial impact on observed disparities between strata.

#### Full model

Next, we entered all individual determinants that define the strata into the model to determine whether the presence of heterogeneity, if any, was due to additive or interactions of effects. This was determined by any change in the VPC_inter_. The VPC for model 6 expresses the degree of the total individual outcome variation having adjusted for the main effects of the variables, which is attributable to multiplicative interaction of effects. The inclusion of the variables will only explain away the between-stratum variance; the within-stratum variance will be unaffected. The reduction in between-stratum variance from null model 1 to full model can also be expressed by the PCV. For this analysis the PCV_inter_ is interpreted as the proportion of the teenage maternity variation between-strata which is attributable to the main effects of the covariates. In contrast, 1 − PCV_inter_ measures the proportion of teenage maternity variation between-strata due to interaction of effects [20] or by the effect of other variables not included in the model. Then, it captures the extent to which interaction effects are necessary to accurately characterize disparities between strata.

Finally, to identify those strata where the observed prevalence was higher/lower than expected based on the additive main effects of the variables that comprise the stratum, residuals and associated 95% CI were obtained. Residuals represent the stratum’s random effects that remain after removing main effects and so capture potential higher-order interactions. Residuals above or below zero represent the excess in the multiplicative scale in the propensity of teenage maternity beyond the additive effect of the variables that comprised the intersectional strata. In the geographic approach, the inclusion of individual variables as fixed effect covariates, removes the differences between departments associated with the differential individual composition of their population. Additional explanation regarding statistics, methodology, and advantage of intersectional MAIHDA to study health inequities can be found elsewhere [21, 23, 34, 35, 49–51].

To provide a practical interpretation of the results and their utility for precision public health [47], we completed the following steps:

1. Identifying a benchmark value: In this case, we selected the worldwide prevalence of 10% as the target to be reached. According to this benchmark, the results can be classified as: A) Target not reached or higher than desired. In this case, the aim of any intervention would be to reduce the prevalence, and B) Target reached. In this case, the aim of any intervention would be to maintain this prevalence level.
2. Quantifying disparity size using VPC: Interpreting VPC as a measure of disparity is the main innovation of MAIHDA compared to conventional studies in inequality. That is, disparities are not measured as differences between group averages but as the share of the total individual differences identified between averages. For the interpretation of our results, we reclassified the above groups as A) Small: <  = 5%, presenting no evidence of disparities, or B) Large: > 5%, presenting evidence of disparities.
3. Creating scenarios to interpret the results: In this step, the information provided above is combined as follows:
  AA: There is no evidence of disparities between subgroups and the target has not been achieved at national level. Therefore, all subgroups have performed similarly badly. Universal interventions are required to reduce adolescent maternity prevalence in the country.
  BA: This is the ideal scenario for the country. There is no evidence for disparities between subgroups and the target has been achieved at national level. Therefore, all subgroups have performed similarly well. Universal interventions are required to maintain the prevalence of adolescent maternity in the country.
  BA: This is the ideal scenario for the country. There is no evidence for disparities between subgroups and the target has been achieved at national level. Therefore, all subgroups have performed similarly well. Universal interventions are required to maintain the prevalence of adolescent maternity in the country.
  AB: There is evidence of disparities. Some subgroups may have achieved the target level even if it has not been achieved at national level. Proportional universalism interventions are then required with the aim of maintaining the prevalence of adolescent maternity in some specific subgroups while reducing it in others.
  BB: There is evidence of disparities. Some subgroups may have not achieved the target level even if it has been achieved at national level. Proportional universalism interventions are then required with the aim of reducing the prevalence of adolescent maternity in some specific subgroups while maintaining it in others.

For scenarios AB and BB, additional steps are needed.

4. Visualizing the predicted prevalence to identify those specific subgroups that might require a different intervention aim (to reduce or maintain the prevalence).
5. Identify the characteristics that contribute most to disparities using partial models to disentangle the mechanisms to be considered in designing a potential intervention.
6. Identify those subgroups with higher-than-expected prevalence because they also need to be prioritized for future studies and targeted interventions.

Two potential C scenarios are also possible where the prevalence is found to be lower-than-expected. This would be a desirable outcome yet, considering the trajectory of the prevalence in the country, under-registration must not be ruled out.

## Results

**Fig. 1:**
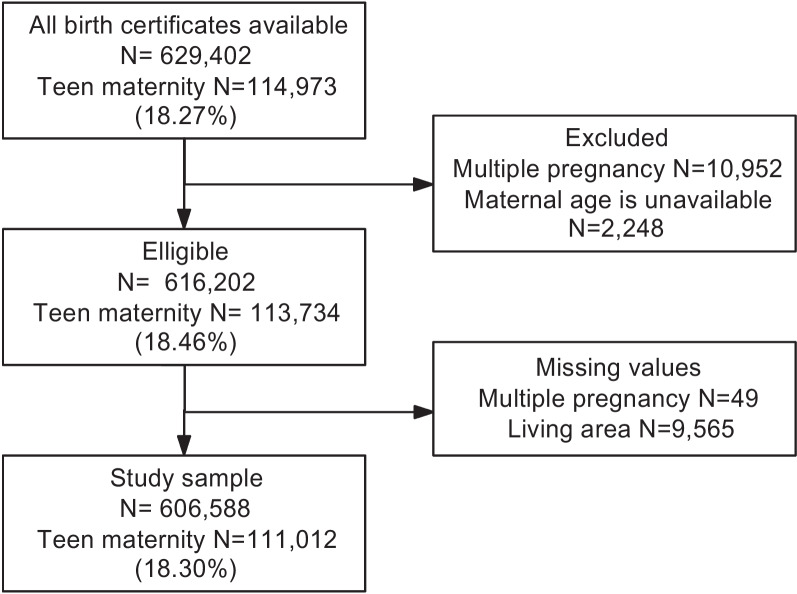
Flow diagram for the study population

**Fig. 2:**
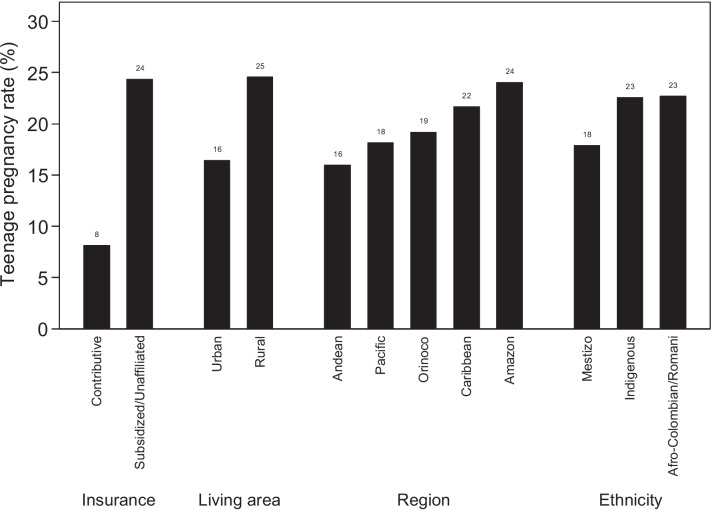
Distribution of teenage maternity according to each socio-geographic determinant. Bars are in ascending ordered by prevalence rate

In 2020, 629,402 live births were registered in Colombia. Figure 1 shows the flow diagram of the study population. The final sample for this study consisted of 606,588 births. Intersectional stratum size varies from 3 to 134,820 mothers. There were 4 strata with less than 20 and 2 strata with less than 10 mothers. The prevalence of teenage mothers was 18.30% (95% CI 18.20–18.40). In the univariate analysis, those belonging to the Subsidized/Unaffiliated insurance group, living in Rural areas, living in the Amazon region, and those of indigenous or Afro-Colombian/Romani ethnicities, showed the highest prevalence compared to their reference categories (Fig. 2, Table 1).

**Table 1:** Description of the study population and prevalence ratio of teenage maternity according to socio-geographic determinants

**Characteristics**	**Total** *N* = 606,588 n(%)	**Age of mother**		**Single level Univariate PRR** (95% CI)	**Single level Multiple PRR** **(95% CI)**
		**Adult** *N*** = **495,576 n(%)	**Teenage** *N* = 111,012 n(%)		
**Healthcare coverage regime**					
Contributive	226,107 (37.28)	207,699 (41.91)	18,408 (16.58)	Ref	
Subsidized/Unaffiliated	380,481 (62.72)	287,877 (58.09)	92,604 (83.42)	2.99 (2.95–3.03)	2.82(2.75–2.87)
**Living area**					
Urban	466,907 (76.97)	390,181 (78.73)	76,726 (69.12)	Ref	
Rural	139,681 (23.03)	105,395 (21.27)	34,286 (30.88)	1.49 (1.48–1.51)	1.21(1.19–1.22)
**Region of residence**					
Andean	298,460 (49.20)	250,831 (50.61)	47,629 (42.90)	Ref	
Amazon	14,657 (2.42)	11,132 (2.25)	3,525 (3.18)	1.51 (1.46–1.55)	1.12(1.09–1.15)
Caribbean	179,216 (29.54)	140,374 (28.33)	38,842 (34.99)	1.36 (1.34–1.37)	1.08(1.07–1.10)
Orinoco	27,009 (4.45)	21,829 (4.40)	5,180 (4.67)	1.20 (1.17–1.23)	1.02(0.99–1.04)
Pacific	87,246 (14.38)	71,410 (14.41)	15,836 (14.27)	1.14 (1.12–1.16)	0.94(0.02–1.95)
**Ethnicity**					
Mestizo	555,621 (91.60)	456,135 (92.04)	99,486 (89.62)	Ref	
Indigenous	27,407 (4.52)	21,223 (4.28)	6,184 (5.57)	1.26 (1.23–1.29)	0.86(0.84–0.88)
Afro-Colombian/Romani	23,560 (3.88)	18,218 (3.68)	5,342 (4.81)	1.27 (1.24–1.30)	1.14(1.11–1,17)

**Fig. 3:**
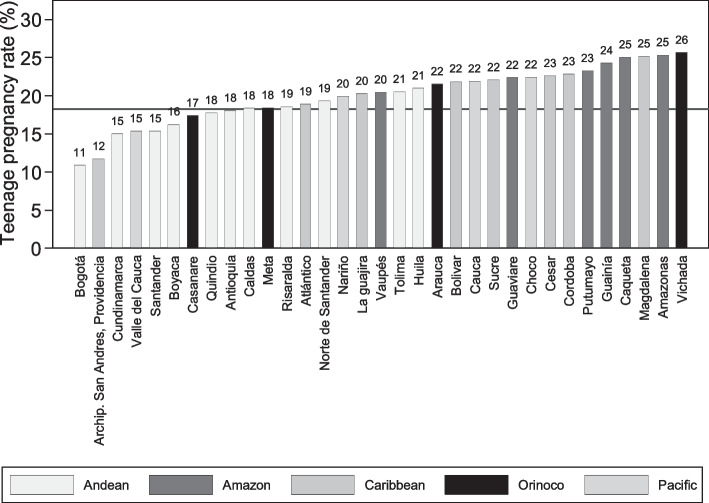
Observed teenage maternity prevalence according to department of residency. The colour of the bar represents which region the department belongs to

The observed prevalence between intersectional strata ranged from 0 to 29% (Table 2). Predicted prevalence based on null multilevel model varies from 6.87% to 29%. All women were adults in the stratum 27, which was the one with the smallest size (Contributive, Afro-Colombian/Romani, Rural, Amazon region, *n* = 3), however their expected prevalence was ~ 15%. Stratum 38 (Subsidized/Unaffiliated, Mestizo, Rural, Caribbean region) showed the highest observed and predicted prevalence (Table 2). Regarding departments, the lowest observed and expected prevalence was detected in Bogotá and the highest in Vichada (Fig. 3, Table 3).

**Table 2:** Distribution of observed and expected teenage mothers’ prevalence according to intersectional socio-geographic strata

Strata	Healthcare coverange regime	Ethnicity	Living area	Region	N total (teen mothers)	Observed Prevalence (95%CI)	Expected Prevalence (95%CI)
1	Contributive	Mestizo	Urban	Andean	134,820 (9,988)	7.41(7.27–7.55)	7.41(7.27–7.56)
2	Contributive	Mestizo	Urban	Amazon	1,745 (132)	7.56(6.41–8.9)	7.73(6.54–9.12)
3	Contributive	Mestizo	Urban	Caribbean	36,071 (2,855)	7.91(7.64–8.2)	7.92(7.64–8.22)
4	Contributive	Mestizo	Urban	Orinoco	7,216 (543)	7.52(6.94–8.16)	7.57(6.96–8.22)
5	Contributive	Mestizo	Urban	Pacific	21,932 (1,528)	6.97(6.64–7.31)	6.98(6.64–7.34)
6	Contributive	Mestizo	Rural	Andean	12,712 (1,863)	14.66(14.05–15.28)	14.66(14.01–15.34)
7	Contributive	Mestizo	Rural	Amazon	194 (17)	8.76(5.51–13.66)	9.74(6.49–14.62)
8	Contributive	Mestizo	Rural	Caribbean	2,370 (452)	19.07(17.54–20.7)	19.03(17.36–20.86)
9	Contributive	Mestizo	Rural	Orinoco	1,026 (108)	10.53(8.79–12.56)	10.67(8.88–12.82)
10	Contributive	Mestizo	Rural	Pacific	2,572 (346)	13.45(12.19–14.83)	13.47(12.13–14.95)
11	Contributive	Indigenous	Urban	Andean	75 (8)	10.67(5.4–19.99)	12.06(7.07–20.56)
12	Contributive	Indigenous	Urban	Amazon	65 (7)	10.77(5.19–21.01)	12.28(7.04–21.4)
13	Contributive	Indigenous	Urban	Caribbean	210 (11)	5.24(2.92–9.22)	6.87(4.38–10.79)
14	Contributive	Indigenous	Urban	Orinoco	12 (3)	25(7.85–56.61)	18.87(8.86–40.22)
15	Contributive	Indigenous	Urban	Pacific	93 (3)	3.23(1.04–9.58)	6.95(3.84–12.58)
16	Contributive	Indigenous	Rural	Andean	95 (14)	14.74(8.9–23.41)	14.84(9.41–23.4)
17	Contributive	Indigenous	Rural	Amazon	28 (6)	21.43(9.81–40.61)	18.64(9.93–35)
18	Contributive	Indigenous	Rural	Caribbean	180 (12)	6.67(3.82–11.39)	8.16(5.21–12.78)
19	Contributive	Indigenous	Rural	Orinoco	23 (3)	13.04(4.16–34.16)	14.55(7.21–29.35)
20	Contributive	Indigenous	Rural	Pacific	122 (15)	12.3(7.53–19.43)	12.87(8.32–19.93)
21	Contributive	Afro-Colombian/Romani	Urban	Andean	780 (74)	9.49(7.62–11.75)	9.72(7.81–12.1)
22	Contributive	Afro-Colombian/Romani	Urban	Amazon	18 (1)	5.56(0.73–31.91)	12.13(5.67–25.98)
23	Contributive	Afro-Colombian/Romani	Urban	Caribbean	478 (45)	9.41(7.1–12.38)	9.80(7.45–12.88)
24	Contributive	Afro-Colombian/Romani	Urban	Orinoco	26 (4)	15.38(5.78–35.02)	15.54(7.93–30.44)
25	Contributive	Afro-Colombian/Romani	Urban	Pacific	2,605 (275)	10.56(9.43–11.8)	10.61(9.44–11.93)
26	Contributive	Afro-Colombian/Romani	Rural	Andean	111 (18)	16.22(10.44–24.32)	15.99(10.55–24.25)
27	Contributive	Afro-Colombian/Romani	Rural	Amazon	3 (0)	0	14.80(6.06–36.13)
28	Contributive	Afro-Colombian/Romani	Rural	Caribbean	137 (16)	11.68(7.27–18.24)	12.33(8.06–18.85)
29	Contributive	Afro-Colombian/Romani	Rural	Orinoco	9 (1)	11.11(1.36–53.13)	14.90(6.59–33.71)
30	Contributive	Afro-Colombian/Romani	Rural	Pacific	379 (60)	15.83(12.49–19.87)	15.77(12.34–20.14)
31	Subsidized/ Unaffiliated	Mestizo	Urban	Andean	102,353 (23,134)	22.6(22.35–22.86)	22.6(22.31–22.89)
32	Subsidized/ Unaffiliated	Mestizo	Urban	Amazon	6,561 (1,715)	26.14(25.09–27.22)	26.10(24.9–27.37)
33	Subsidized/ Unaffiliated	Mestizo	Urban	Caribbean	97,209 (24,207)	24.9(24.63–25.17)	24.90(24.59–25.22)
34	Subsidized/ Unaffiliated	Mestizo	Urban	Orinoco	11,742 (2,744)	23.37(22.61–24.14)	23.35(22.49–24.24)
35	Subsidized/ Unaffiliated	Mestizo	Urban	Pacific	23,605 (4,785)	20.27(19.76–20.79)	20.27(19.7–20.85)
36	Subsidized/ Unaffiliated	Mestizo	Rural	Andean	43,684 (11,482)	26.28(25.87–26.7)	26.28(25.8–26.8)
37	Subsidized/ Unaffiliated	Mestizo	Rural	Amazon	3,501 (993)	28.36(26.89–29.88)	28.28(26.58–30.1)
38	Subsidized/ Unaffiliated	Mestizo	Rural	Caribbean	27,873 (8,105)	29.08(28.55–29.61)	29.07(28.44–29.71)
39	Subsidized/ Unaffiliated	Mestizo	Rural	Orinoco	4,782 (1,173)	24.53(23.33–25.77)	24.48(23.12–25.92)
40	Subsidized/ Unaffiliated	Mestizo	Rural	Pacific	13,653 (3,316)	24.29(23.58–25.01)	24.27(23.46–25.11)
41	Subsidized/ Unaffiliated	Indigenous	Urban	Andean	315 (79)	25.08(20.59–30.17)	24.4(19.63–30.32)
42	Subsidized/ Unaffiliated	Indigenous	Urban	Amazon	754 (199)	26.39(23.37–29.66)	26.07(22.7–29.93)
43	Subsidized/ Unaffiliated	Indigenous	Urban	Caribbean	3,288 (728)	22.14(20.75–23.59)	22.09(20.54–23.75)
44	Subsidized/ Unaffiliated	Indigenous	Urban	Orinoco	271 (74)	27.31(22.32–32.93)	26.39(21.07–33.05)
45	Subsidized/ Unaffiliated	Indigenous	Urban	Pacific	1,141 (216)	18.93(16.76–21.31)	18.84(16.5–21.51)
46	Subsidized/ Unaffiliated	Indigenous	Rural	Andean	2,102 (610)	29.02(27.12–31)	28.88(26.68–31.26)
47	Subsidized/ Unaffiliated	Indigenous	Rural	Amazon	1,706 (441)	25.85(23.83–27.98)	25.71(23.42–28.22)
48	Subsidized/ Unaffiliated	Indigenous	Rural	Caribbean	8,702 (1,707)	19.62(18.8–20.46)	19.6(18.69–20.55)
49	Subsidized/ Unaffiliated	Indigenous	Rural	Orinoco	1,838 (510)	27.75(25.75–29.84)	27.6(25.31–30.1)
50	Subsidized/ Unaffiliated	Indigenous	Rural	Pacific	6,387 (1,538)	24.08(23.05–25.14)	24.05(22.88–25.28)
51	Subsidized/ Unaffiliated	Afro-Colombian/Romani	Urban	Andean	918 (224)	24.4(21.73–27.29)	24.17(21.22–27.53)
52	Subsidized/ Unaffiliated	Afro-Colombian/Romani	Urban	Amazon	39 (5)	12.82(5.38–27.57)	13.99(7.51–26.08)
53	Subsidized/ Unaffiliated	Afro-Colombian/Romani	Urban	Caribbean	1,962 (511)	26.04(24.15–28.03)	25.92(23.77–28.26)
54	Subsidized/ Unaffiliated	Afro-Colombian/Romani	Urban	Orinoco	44 (12)	27.27(16.08–42.32)	23.18(13.89–38.68)
55	Subsidized/ Unaffiliated	Afro-Colombian/Romani	Urban	Pacific	10,559 (2,616)	24.78(23.96–25.61)	24.75(23.82–25.72)
56	Subsidized/ Unaffiliated	Afro-Colombian/Romani	Rural	Andean	495 (135)	27.27(23.53–31.37)	26.76(22.63–31.64)
57	Subsidized/ Unaffiliated	Afro-Colombian/Romani	Rural	Amazon	43 (9)	20.93(11.18–35.76)	18.87(10.85–32.8)
58	Subsidized/ Unaffiliated	Afro-Colombian/ Romani	Rural	Caribbean	736 (193)	26.22(23.17–29.52)	25.89(22.5–29.8)
59	Subsidized/ Unaffiliated	Afro-Colombian/Romani	Rural	Orinoco	20 (5)	25(10.55–48.5)	19.87(10.12–39.03)
60	Subsidized/ Unaffiliated	Afro-Colombian/Romani	Rural	Pacific	4,198 (1,138)	27.11(25.78–28.47)	27.05(25.52–28.66)

**Table 3:** Distribution of observed and expected teenage mothers’ prevalence according to department of residency

**Geopolitical Department of residence**	**Total** *N* = 606.588 n(%)	**Adult** *N*** = **495.576 n(%)	**Adolescent** *N* = 111.012 n(%)	**Observed Prevalence (95%CI)**	**Expected Prevalence** (95% CI)
Bogotá	77.801 (12.83)	69.301 (13.98)	8.500 (7.66)	10.92(10.70–11.15)	10.94(10.72–11.18)
Antioquia	70.483 (11.62)	57.740 (11.65)	12.743 (11.48)	18.08(17.79–18.36)	18.08(17.77–18.4)
Atlántico	39.285 (6.48)	31.860 (6.43)	7.425 (6.69)	18.90(18.52–19.29)	18.90(18.48–19.34)
Bolivar	32.786 (5.40)	25.611 (5.17)	7.175 (6.46)	21.88(21.44–22.34)	21.87(21.37–22.39)
Boyaca	13.150 (2.17)	11.012 (2.22)	2.138 (1.93)	16.26(15.64–16.90)	16.29(15.62–16.99)
Caldas	7.807 (1.29)	6.370 (1.29)	1.437 (1.29)	18.41(17.56–19.28)	18.42(17.5–19.39)
Caqueta	6.123 (1.01)	4.589 (0.93)	1.534 (1.38)	25.05(23.98–26.15)	24.94(23.73–26.21)
Cauca	17.505 (2.89)	13.670 (2.76)	3.835 (3.45)	21.91(21.30–22.53)	21.89(21.21–22.59)
Cesar	22.218 (3.66)	17.191 (3.47)	5.028 (4.53)	22.63(22.08–23.18)	22.61(22–23.24)
Cordoba	24.353 (4.01)	18.784 (3.79)	5.569 (5.02)	22.87(22.34–23.40)	22.85(22.26–23.46)
Cundinamarca	34.249 (5.65)	29.100 (5.87)	5.149 (4.64)	15.03(14.66–15.42)	15.05(14.65–15.47)
Choco	7.721 (1.27)	5.987 (1.21)	1.734 (1.56)	22.46(21.54–23.40)	22.41(21.38–23.48)
Huila	16.568 (2.73)	13.082 (2.64)	3.486 (3.14)	21.04(20.43–21.67)	21.03(20.34–21.73)
La Guajira	22.457 (3.70)	17.897 (3.61)	4.560 (4.11)	20.30(19.78–20.84)	20.30(19.72–20.9)
Magdalena	23.550 (3.88)	17.609 (3.55)	5.941 (5.35)	25.23(24.68–25.79)	25.20(24.57–25.84)
Meta	15.138 (2.50)	12.344 (2.49)	2.794 (2.52)	18.46(17.85–19.08)	18.46(17.8–19.16)
Nariño	16.091 (2.65)	12.885 (2.60)	3.206 (2.89)	19.92(19.31–20.54)	19.92(19.24–20.62)
Norte de Santander	21.699 (3.58)	17.506 (3.53)	4.193 (3.78)	19.32(18.80–19.85)	19.32(18.75–19.92)
Quindío	5.353 (0.88)	4.402 (0.89)	951 (0.86)	17.76(16.76–18.81)	17.81(16.73–18.96)
Risaralda	9.873 (1.63)	8.037 (1.62)	1.836 (1.65)	18.60(17.84–19.37)	18.61(17.78–19.47)
Santander	25.768 (4.25)	21.798 (4.40)	3.970 (3.58)	15.41(14.97–15.85)	15.43(14.96–15.92)
Sucre	13.825 (2.28)	10.768 (2.17)	3.057 (2.75)	22.11(21.43–22.81)	22.09(21.32–22.88)
Tolima	15.709 (2.59)	12.483 (2.52)	3.226 (2.91)	20.54(19.91–21.17)	20.53(19.83–21.24)
Valle del Cauca	45.929 (7.57)	38.868 (7.84)	7.061 (6.36)	15.37(15.05–15.71)	15.39(15.03–15.75)
Arauca	4.102 (0.68)	3.217 (0.65)	885 (0.80)	21.57(20.34–22.86)	21.5(20.15–22.95)
Casanare	6.002 (0.99)	4.955 (1.00)	1.047 (0.94)	17.44(16.50–18.42)	17.49(16.47–18.56)
Putumayo	4.553 (0.75)	3.492 (0.70)	1.061 (0.96)	23.30(22.09–27.55)	23.2(21.85–24.62)
San Andres. Providencia and Santa Catalina	741 (0.12)	654 (0.13)	87 (0.08)	11.74(9.61–14.27)	13.14(11.02–15.67)
Amazonas	1.057 (0.17)	789 (0.16)	268 (0.24)	25.35(22.82–28.07)	24.73(22.03–27.76)
Guainía	1.024 (0.17)	775 (0.16)	249 (0.22)	24.32(21.78–27.04)	23.78(21.1–26.79)
Guaviare	1.245 (0.21)	966 (0.19)	279 (0.25)	22.41(20.18–24.81)	22.12(19.76–24.76)
Vaupés	655 (0.11)	521 (0.11)	134 (0.12)	20.46(17.54–23.72)	20.27(17.36–23.67)
Vichada	1.767 (0.29)	1.313 (0.26)	454 (0.41)	25.69(23.71–27.78)	25.29(23.11–27.67)

Table 4 presents the results from the multilevel models. Model 1 shows the contextual effect of intersectional strata was moderate (VPC_null_logit_ = 9.16%). The proportion of outcome variation lying within strata was 95.39%. Partial models suggested that health insurance reduced the between-strata variance by PCV_partial_logit_ = 83.32%, while region of residency only reduced the between-strata variance by PCV_partial_logit_ = 1.37%. The full intersectional model showed that around 90% of the variation between-strata was due to the main effects of the variables that defined the strata (PCV_full_logit_ = 90%), and 10% was due to two- and higher-way multiplicative interaction between the variables comprising the strata.

**Table 4:** Geographical and intersectional multilevel models of teenage maternity in Colombia in 2020

Analysis	Intersectional-Socio-geographical						Geographical
**Models**	**Null**	**Partials**				**Full**	**Null**
	**Model 1**	**Model 2 Healthcare Coverage Regime**	**Model 3 Urbanicity**	**Model 4** **Region**	**Model 5** **Ethnicity**	**Model 6** **Intersectional Full**	**Model 7** **Department Null**
***Variance Poisson***	.231 (.169- .314)	.039(.021-.071)	.213(.162-.281)	.228(.168-.309)	.227(.166-.310)	.023(.013- .042)	.037 (.020- .072)
***AUC Poisson***	64.65 (64.48- 64.82)	64.65(64.48–64.81)	64.65(64.48–64.81)	64.65(64.48–64.82)	64.65(64.49–64.82)	64.64 (64.48- 64.81)	57.38(57.20–57.56)
***VPC Poisson (%) (Leckie)***	4.61	0.78	3.97	4.57	4.81	0.43	0.76
***PCV Poisson (%)***	Reference	82.99	7.58	1.12	5.39	90.01	Reference
***BIC***	693.9	618.1	694.3	709.4	1150.5	625.4	494.4047
***Variance logit***	.332(.221-.497)	.055(.034-.088)	.308(.204-.463)	.344(.232-.508)	.327(.218-.492	.032 (.019-.052)	.057(.034-.094)
***VPC Logit (%)***	9.16	1.65	8.55	9.45	9.04	0.95	1.70
***PCV logit (%)***	Reference	83.32	7.19	3.64	1.37	90.44	Reference
Health Insurance							
Contributive	*Reference*						
Subsidized/Unaffiliated	**-**	2.44(2.08–2.89)				2.41(2.15–2.71)	-
Urbanicity							
Urban	*Reference*						
Rural	**-**		1.29(1–1.66)			1.28(1.15–1.41)	-
Region							
Andean	*Reference*						
Amazon	**-**			0.94(0.62–1.42)		0.97(0.83–1.13)	-
Caribbean	**-**			0.89(0.59–1.34)		0.96(0.82–1.12)	-
Orinoco	**-**			1.04(0.70–1.54)		0.96(0.84–1.10)	-
Pacific	**-**			0.89(0.60–1.30)		0.92(0.83–1.03)	-
Ethnicity							
Mestizo	*Reference*						
Indigenous	**-**				1.06(0.75–1.50)	0.97(0.86–1.08)	-
Afro-Colombian / Romani	**-**				1.08 (0.80–1.47)	1.08(0.98–1.21)	-

**Fig. 4:**
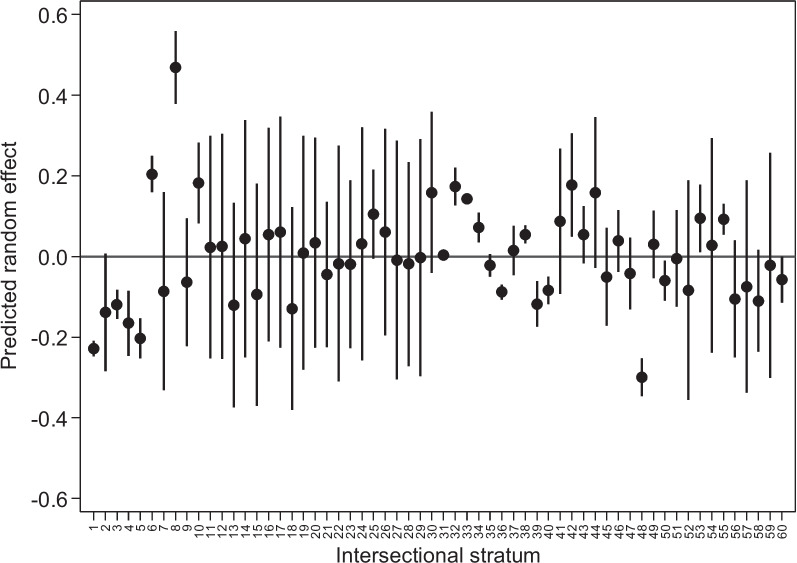
Predicted random effect for each intersectional strata based on the full Poisson regression model

Overall, 30% of strata showed interactions of effects (18 out 0f 60). The distribution of positive and negative residuals is presented in Fig. 4. We found 9 strata with lower-than-expected prevalence and 9 strata with higher-than-expected prevalence. Stratum 8 (Contributive, Mestizo, Rural, Caribbean) and Stratum 48 (Subsidized/Unaffiliated, Indigenous, Rural, Caribbean) showed the most positive and most negative deviations from expected prevalence, respectively.

Just 1% of the variation in the propensity for teenage maternity was between departments and 99% was within departments (VPC_null_logit_ = 1.7%). Therefore, the further steps for the geographic differences in the propensity of teenage maternity were unnecessary for the purposes of this study.

## Discussion

Teenage maternity continues to be a public health concern in Colombia. Rather than reporting on how prevalence varies by a single variable at a time, we combined social and geographical data in an intersectional MAIHDA analysis. We showed that intersectional socio-geographical differences, and therefore disparities, in teenage maternity were much larger than geographical differences based on geopolitical departments. While some intersectional strata may be protective, others may increase individual risk, and mapping these intersectional strata heterogeneity is of fundamental relevance in public health. We illustrated how MAIHDA can be used to evaluate social disparities in teenage maternity prevalence.

The prevalence in this study was 18.3%, higher than the 17.4% reported in Colombia in 2015 [12] and the 10.3% reported worldwide [2]. Teenage pregnancy prevalence is likely to be even higher considering that around 55% of unintended pregnancies in teenagers between 15–19 years end in abortion in low to middle income countries [52]. At the time of conducting this study, abortion remained illegal in Colombia except in cases considered to be life-threatening for the mother which, together with the predominance of religious faith and the increased social status that motherhood obtains according to women, especially in rural areas [17], might contribute to the high prevalence of teenage pregnancies. As expected, the categories with the highest prevalence of teenage maternity were: Subsidized/Unaffiliated in reference to healthcare insurance coverage regime, living in rural areas, living in the Amazon region, and belonging to Indigenous or Afro-Colombian ethnicities. These categories also represent the populations with the lowest socioeconomic status which in turn has been associated with poverty, lower education levels, earlier sexual intercourse, school dropout, and reduced information about contraception [53]. It has been reported that 69.9% of women aged 15–19 in Colombia do not use any form of contraception, and only 59.5% of those aged 13–49 are aware that public health care centers provide free contraceptives [54].

The social variable with the highest impact on between-strata variation (higher PCV_partial_) was healthcare insurance affiliation. The observed and predicted prevalences were higher in intersectional strata with this characteristic (strata range: 13.99%-29.07%) when compared to those strata where women had a contributive affiliation (strata range: 8.87%-19.03%). These disparities in healthcare coverage regime, are in line with previous results related to teenage maternity in Colombia [18] and other sexual and reproductive health indicators [55]. But none of them have integrated intersectionality for a more precise understanding of the disparities. Measures of association alone unable to identify stratum 8 where the prevalence of teenage maternity was even higher than expected based on main additive effect, despite this stratum includes women belonging to the contributive system. Research is needed into whether the quality of Youth Friendly Health Services varies significantly between healthcare coverage regimes and its potential impact on teenage maternity, or even whether this strategy might work for some subgroups in Colombia but needs to be redesigned for others.

For those living in rural areas, the cost of transportation to a healthcare service provider where sexual health and reproductive health programs are provided might explain the increased prevalence of teenage pregnancy in this population when compared to urban areas, where most health services exists [56–58]. A previous systematic review in Africa also shows rural residing as a risk factor for teenage maternity [59], however the included studies used single level analysis. Our research provide evidence reporting measures of associations alone are insufficient to make public health decision i.e., some specific urban areas might also require attention, for example those belonging to the Subsidized/ Unaffiliated and Mestizo category. It has been previously reported that complex social processes arise in rural areas in Colombia including, but not limited to, the exposure of communities to armed conflict, sexual violence, and social role assigned to women within a more patriarchal culture with highly defined gender roles [25].

The Mestizo category showed the lowest prevalence of teenage maternity compared to the Indigenous and Afro-Colombian/Romani categories in the univariate analysis, which is in line with previous reports [18, 60]. Interestingly, Indigenous and Afro-Colombian ethnicities showed a lower or even no association after covariate adjustment. Indeed, the stratum with the lowest prevalence was 15 (Contributive, Indigenous, Urban area, Pacific region). Ethnicity and the Region of residence did not substantially reduce the between-stratum variance as shown by the lower PCV_partial_ values. Despite this, ethnicity has been reported as a source of social disparities at population-level, these results question the use of ethnicity alone for individual-level risk experience profiling in Colombia.

Socio-geographical intersectional strata variation was moderate (VPC_null-logit_ > 5) but lower than previous studies with different outcomes [61, 62]. The moderate intersectional-VPC as a measure of social disparity shows how different patterns of disadvantage generated by multiple and compounded dimensions of socio-economic variables posit different risks for adolescent maternity. For example, among the strata comprised by Contributive healthcare regime and ‘Mestizo’ ethnicity, a vis-a-vis comparison between strata from the same region shows that the prevalence of teenage maternity is lower in Urban areas (strata 1–5) than in women residing in Rural areas (strata 6–10). However, the magnitude of the effect of rurality is influenced by context. For example, the prevalence in stratum 2 (Urban, Amazon) was similar to the prevalence in stratum 7 (Rural, Amazon); in contrast, the prevalence in stratum 8 (Rural, Caribbean) was much higher than the prevalence in stratum 3 (Urban, Caribbean), while setting them equal to the other categories that comprised the strata.

Interestingly, 90% of the variation in teenage maternity between-strata was due to additive effects and 10% due to interaction effects or by the effects of other variables not included in the model. Positive and negative specific interactions of effects were observed at the strata levels. Interestingly, stratum 1 and 5 showed lower-than-expected prevalence, while stratum 6 and 8 showed higher-than-expected prevalence, with all the data within these strata included the category of contributive healthcare regime. In contrast, stratum 48, including those belonging to the Subsidized/Unaffiliated healthcare insurance category showed the lower-than-expected prevalence. Overall, these results show that the univariate social gradient associated with one factor might be lost when they are balanced by the effects of other factors. Therefore, analyzing social disparities using one factor at a time might distort results and provide misleading information, potentially resulting in the stigmatization of social groups. From a public health perspective, our results show one-size might not-fits-all when promoting social equity in the reproductive health in Colombia.

The observed prevalence between Colombia’s geographical departments varies from 11% in the capital district of Bogota to 26% in the department of Vichada in the country’s Orinoquía region. However, this inter-departmental variation masks a far higher variation within departments, meaning that identifying the department where a woman lives says relatively little about her risk of experiencing a teenage motherhood. Similar findings were obtained at municipal level in a previous study in 2015 [18], and even greater disparities in teenage pregnancy based on location was observed at community level in Ethiopia [63] and Zambia [64]. Our results, using departments as second level and in a context of high prevalence indicates that universal intervention might be required to reduce teenage prevalence in Colombia. However, results from the intersectional analysis suggest that a proportional universalism approach would potentially be more cost-effective.

Previous studies have used multilevel analysis to identify the effects of individual and contextual variables [18, 64–66] without considering VPC as a measure of GCE. Others have focused on differences between group means [59, 67] without considering teenage heterogeneity around them, which may lead to the attribution of the same risk to all women within a stratum [68], thereby disregarding how interlocking factors can produce varying levels of advantage/protection or disadvantage/risk in the study outcome. In this regard, and to the best of our knowledge, this is the first study using MAIHDA to examine public health disparities for teenage maternity taking an intersectional perspective. Under the MAIHDA strategy, all strata are of interest even the “reference” category. This approach has recently gained attention [21, 36, 50, 51, 69, 70] as an effective tool to study social inequalities.

A stratified analysis by intersectional strata can be considered a strategy to control for confounders. However, the aim of stratification here was to describe outcome heterogeneity across intersectional strata, to understand how the burden of the outcome is distributed across population groups. This is known as descriptive intersectional-MAIHDA. We also performed an analytical intersectional-MAIHDA by performing partially and fully adjusted MAIHDA models to investigate potential causes driving outcome disparities [71]. In this case, variables were included as fixed effect covariates as in classical analytical single-level confounder adjustment. However, the aim in MAIHDA was not to estimate the independent effect of the variables, but to remove their additive effect from the outcome heterogeneity.

There are evident limitations to our study. First, there are likely additional factors both influencing our outcome and acting as confounders or potential mediators, but which are not available through the information on the birth certificate, for example, income, contraception use, sexual education, or sexual abuse. Although all the women in the study were residing in Colombia, inferring a high likelihood they were Colombian, nationality was not explicitly documented in the dataset. Future research integrating these variables are warranted to provide a more comprehensive analysis of intersectionality [72] and to prioritize where and how to intervene to reduce this public health problem.

Second, a misclassification bias of ethnicity could be present in our data as we used a proxy on the mother’s ethnicity based on data from the child. A person may give a different response regarding their ethnicity depending on the context [73]. This could be the case when parents are classifying their children at birth, for example, considering that in Colombia, racism, ignorance about the cultural contributions of Africa and black heritage to the country might contribute to misclassification. Some Afrocolombians may have classified themselves as “mestizo” rather than Afrocolombian, as the prevalence of newborns categorized as Afrocolombian in the birth certificate data was lower than expected, given national level data on ethnicity.

Third, the categorization based on region can be seen as simplistic and insufficient, as it disregards the large heterogeneities within each region. One may argue that, for the intersectional analysis, departments should have been used rather than regions to provide a greater disaggregation of strata. However, it is also important to balance that this would lead to a very high number of strata (i.e., 2 × 3 × 2 × 33 = 396 strata), many of which, due to their smaller size, would get shrunk more towards the overall prevalence due to shrinkage. Despite the advantage of shrunken prediction in MAIHDA, predicted stratum-specific prevalence and interaction effects for small strata can be still uncertain; this could be specially the case for some strata including indigenous or afrocolombian/romanies women. Therefore, we need to balance the benefits of using broader categories i.e. regions, with the substantive utility of this approach where we want the individual stratum to be substantively interesting and meaningful. Some multi-categorical strata were rather small, which is reflected in the wide CIs, generating limited reliability for some point estimates. In a sensitivity analysis, the exclusion of strata with sample sizes < 10 did not substantially change the model results.

Fourth, there were missing data regarding the area of residency that were positively related with outcome, therefore, the estimated proportion of adolescent maternity for strata containing town/rural areas might be underestimated and the magnitude of disparities could be even higher than that reported. Finally, the study analyzed data gathered during the Covid-19 pandemic, and it is possible that some results will vary when studying the years that follow. The lockdown started in Colombia in March 2020, therefore effects on the study’s results could well be possible by the end of 2020.

## Conclusion

The prevalence of teenage maternity in Colombia is higher than that reported worldwide. Using VPC as a summary measure of GCE, we did not observe disparities between Colombia’s geographical departments. All departments showed a higher prevalence than the worldwide average and while there were some variations in prevalences these were somewhat limited. Based on this geographical approach, universal interventions in Colombia would be required to face the problem. By contrast, we observed far greater disparities under the socio-geographical intersectional approach. The identification of patterns or complex combinations of socio-geographical characteristics could inform policies targeting specific intersectional groups that could result in a more effective decline in adolescent pregnancy, following the proportional universalism strategy.

Overall, our results indicate that if resources for prevention are limited, using an intersectional socio-geographical approach might be more effective than implementing universal interventions across all departments. More specifically, strategies focusing on the Subsidized/Unaffiliated healthcare coverage regime, which contributed the most to adolescent maternity disparities, and in strata with a higher prevalences and larger strata sizes, for example strata 31–40, or in those strata with unexpectedly higher prevalences would be a more targeted and cost-effective. We encourage the use of VPC as a measure of inequity in future studies and to explore other potential factor combinations, for example nationality or migration status, and to better understand the potential interactive mechanisms that were observed in some groups, for example in stratum 8 and stratum 48. Our results underscore the need to use an intersectional approach to map health disparities and to minimize the risk of unfounded stigmatization of some social groups.

### Supplementary Information

**Supplementary material 1.**

## Data Availability

The dataset analyzed during the current study was obtained from Departamento Administrativo Nacional de Estadísticas www.dane.gov.co

## Abbreviations

AUC: Area under the Curve
CI: Confidence Interval
DANE: National Administrative Department of Statistics
GCE: General Contextual Effect
MAIHDA: Multilevel Analysis of Individual Heterogeneity and Discriminatory Accuracy
PCV: Proportional Change in Variance
PRR: Prevalence Rate Ratio
SISBEN: System for the Identification of Potential Beneficiaries of Social Programs
VPC: Variance Partition Coefficient
