# Supplementary material for: Mapping socio-geographical disparities in the occurrence of teenage maternity in Colombia using multilevel analysis of individual heterogeneity and discriminatory accuracy (MAIHDA)
Source: Int J Equity Health. 2024 Feb 23;23:36. doi: 10.1186/s12939-024-02123-5 (PMC10885464; doi:10.1186/s12939-024-02123-5)
Supplement: Supplementary file 1 — Supplementary material 1. [file 12939_2024_2123_MOESM1_ESM.pdf]

```
*****************************************************************************************

* MANUSCRIPT Mapping socio-geographical disparities in the occurrence of teenage maternity in Colombia

//using multilevel analysis of individual heterogeneity and discriminatory accuracy (MAIHDA)

*****************************************************************************************


version 14


clear *


set cformat %9.3f



*****************************************************************************************

* (1) PREPARE THE DATA

*****************************************************************************************


* Directory

cd "Y:\manuscripts-2-in-progress"


* Load the collapsed data

use "teentoc.dta", clear




* Order variables

order departo inter id insurance livingarea region etnia matage


* Recode inter as there is a gap at 1 in the integer sequence from 0 to 60

distinct inter
```

```
assert r(ndistinct) ==  60
summarize inter
assert r(min) == 0
assert r(max) == 60
assert inter != 1
recode inter (0 = 1)
distinct inter
assert r(ndistinct) ==  60
summarize inter
assert r(min) == 1
assert r(max) == 60

* Label variables
label var inter "Intersectional stratum"

* Attach value label on insurance
tabulate insurance, missing
describe insurance
label list insurance
label define insurance ///
  0 "Contributive" ///
  1 "Subsidized/No affiliation" ///
  , replace
label values insurance insurance
tabulate insurance, missing

* Attach value label on livingarea
tabulate livingarea, missing
describe livingarea
```

```
label list livingarea
label define livingarea ///
  0 "Urban" ///
  1 "Rural" ///
  , replace
label values livingarea livingarea
tabulate livingarea, missing

* Attach value label on etnia
tabulate etnia, missing
describe etnia
label list etnia
label define etnia ///
  0 "Mestizo" ///
  1 "Indigenous" ///
  2 "Afro-Colombian/Romani" ///
  , replace
label values etnia etnia
tabulate etnia, missing

* Compress the data
compress

* Save the data
save "teentoc_clean.dta", replace


*------------------------------------------------------------------------------
* Collapse the data down to the stratum level and save
```

```
*------------------------------------------------------------------------------

* Load the data
use "teentoc_clean.dta", replace

* Collapse the data down to one observation per stratum
collapse (sum) y = matage (count) n = matage (mean) p = matage, by(inter insurance livingarea
region etnia)

* Format the variables
format %3.2f p

* Compress the data
compress

* Save the data
save "teentoc_clean_inter.dta", replace


*------------------------------------------------------------------------------
* Collapse the data down to the department level and save
*------------------------------------------------------------------------------

* Load the data
use "teentoc_clean.dta", replace

* Collapse the data down to one observation per department
collapse (sum) y = matage (count) n = matage (mean) p = matage, by(departo)
```

```
* Format the variables
format %3.2f p

* Compress the data
compress

* Save the data
save "teentoc_clean_departo.dta", replace



*------------------------------------------------------------------------------
* Collapse the data down to the department-by-stratum level and save
*------------------------------------------------------------------------------

* Load the data
use "teentoc_clean.dta", replace

* Collapse the data down to one observation per stratum
collapse (sum) y = matage (count) n = matage (mean) p = matage, by(departo inter insurance
livingarea region etnia)

* Format the variables
format %3.2f p

* Compress the data
compress

* Save the data
save "teentoc_clean_departo_inter.dta", replace
```

```
******************************************************************************
* (2) DESCRIPTIVE STATISTICS
******************************************************************************

* Load the data
use "teentoc_clean.dta", clear

*-----------------------------------------------------------------------------
* Univariate descriptive statistics for mother characteristics
*-----------------------------------------------------------------------------
* Tabulate the dependent variable
proportion matage

* Tabulate departments
tabulate departo

* Tabulate strata
tabulate inter

* Tabulate each explanatory variable
tabulate insurance
tabulate livingarea
tabulate region
tabulate etnia


*-----------------------------------------------------------------------------
```

```
* Bivariate descriptive statistics for mother characteristics
*-----------------------------------------------------------------------------
proportion matage, over(departo)
proportion matage, over(inter)

tabulate departo matage, col chi2
tabulate insurance matage, col chi2
tabulate livingarea matage, col chi2
tabulate region matage, col chi2
tabulate etnia matage, col chi2

* Univariate Poisson regression
xi: poisson matage i.departo, vce(robust) irr difficult
xi: poisson matage i.insurance, vce(robust) irr difficult
xi: poisson matage i.livingarea, vce(robust) irr difficult
xi: poisson matage i.etnia, vce(robust) irr difficult
xi: poisson matage i.region, vce(robust) irr difficult
xi: poisson matage i.inter, vce(robust) irr difficult

* Multiple Poisson regression
xi: poisson matage i.insurance i.livingarea i.etnia i.region, vce(robust) irr difficult



*-----------------------------------------------------------------------------
* Figure of mother characteristic rates
*-----------------------------------------------------------------------------

* Save stratum variable specific datasets storing the proportion in each category
foreach var in insurance livingarea region etnia {
```

```
        use "teentoc_clean.dta", clear
        replace matage = 100*matage
        collapse (mean) matage, by(`var')
        format %9.2f matage
        generate variable = "`var'"
        order variable
        decode `var', generate(category)
        order category, after(variable)
        drop `var'
        compress
        save "`var'.dta", replace
}

clear
generate varorder = .
append using "insurance.dta"
replace varorder = 1 if varorder == .
append using "livingarea.dta"
replace varorder = 2 if varorder == .
append using "region.dta"
replace varorder = 3 if varorder == .
append using "etnia.dta"
replace varorder = 4 if varorder == .
label define varorder ///
        1 "Insurance" ///
        2 "Living area" ///
        3 "Region" ///
        4 "Ethnicity"
label values varorder varorder
```

```
graph bar (asis) matage, ///
	over(category, sort(matage) label(labcolor("black") angle(vertical) labsize(*.7))) ///
	over(varorder, label(labsize(*.8))) ///
	nofill ///
	bar(1, fcolor(black) lcolor(black)) ///
	blabel(bar, size(tiny) format(%3.0f)) ///
	ytitle("Teenage pregnancy rate (%)") ///
	ylabel(0(5)30, angle(horizontal) format(%3.0f) nogrid glcolor()) ///
	scheme(s1mono)
* Export graph
graph save "Figure2.gph", replace
graph export "Figure2.png", replace width(1000)


*-------------------------------------------------------------------------------
* Figure of stratum rates
*-------------------------------------------------------------------------------

* Load the data
use "teentoc_clean.dta", clear

* Multiple matage by 100 so that plot shows as percent
replace matage = 100 * matage

* Plot figure
graph bar (mean) matage, ///
	over(inter, sort(matage) label(labcolor("black") angle(vertical) labsize(*.5))) ///
	bar(1, fcolor(black) lcolor(black)) ///
```

```
            blabel(bar, size(tiny) format(%3.0f)) ///
            ytitle("Teenage pregnancy rate (%)") ///
            ylabel(0(5)30, angle(horizontal) format(%3.0f) nogrid glcolor()) ///
            scheme(s1mono) ///
            b1title("Intersectional stratum")

* Export graph
graph save "Figure3.gph", replace
graph export "Figure3.png", replace width(1000)



*------------------------------------------------------------------------------
* Figure of department rates
*------------------------------------------------------------------------------


* Load the data
use "teentoc_clean.dta", clear


* Multiple matage by 100 so that plot shows as percent
replace matage = 100 * matage


* Calculate the mean of matage
summarize matage


* Plot figure
graph bar (mean) matage, ///
            over(region, sort(matage) label(angle(ninety))) ///
            over(departo, sort(matage) label(angle(ninety) labsize(*.7))) nofill asyvars ///
            bar(1, fcolor(gs15) lcolor(none)) ///
```

```
	bar(2, fcolor(gs6) lcolor(none)) ///
	bar(3, fcolor(gs12) lcolor(none)) ///
	bar(4, fcolor(black) lcolor(none)) ///
	bar(5, fcolor(gs13) lcolor(none)) ///
	blabel(bar, size(vsmall) format(%3.0f)) ///
	ytitle(`"Teenage pregnancy rate (%)"') ///
	yline(18.30105) ///
	ylabel(0(5)30, angle(horizontal) format(%3.0f) nogrid) ///
	legend(rows(1) size(*.7) position(6) span) ///
	scheme(s1mono)

* Export graph
graph save "Figure4.gph", replace
graph export "Figure4.png", replace width(1000)



********************************************************************************
* (1) MAIHDA Intersectional
********************************************************************************

*------------------------------------------------------------------------------
* Model 1 null
*------------------------------------------------------------------------------

* Load the stratum level data
use "teentoc_clean_inter.dta", clear

* Fit the model
mepoisson y, exposure(n) || inter:, vce(robust) irr
```

```
* Store the estimation results
estimates store m1

* Display information criteria
estat ic

* Predict the number of teenage mothers
predict m1n, mu
generate m1ir = m1n/n

* Calculate the ROC
expand 2
bysort inter: generate y01 = _n - 1
assert inlist(y01, 0, 1)
generate int n01 = .
replace n01 = n - y if y01 == 0
replace n01 = y if y01 == 1
roctab y01 m1ir [fweight = n01], summary graph
drop y01 n01
duplicates drop

* Calculate the VPC (via Leckie et al. 2020 method)
predict m1xbhat, xb
replace m1xbhat = m1xbhat - ln(n)
scalar m1sigma2u = _b[var(_cons[inter]):_cons]
generate m1mu = exp(m1xbhat + m1sigma2u/2)
generate m1vpc = ((m1mu^2) * (exp(m1sigma2u) - 1)) / ((m1mu^2) * (exp(m1sigma2u) - 1) +
m1mu)
```

```
summarize m1vpc [fweight = n]

* Calculate the VPC (via the latent response formulation of melogit)
meqrlogit y || inter:, binomial(n)
scalar m1logitsigma2u = exp(_b[lns1_1_1:_cons])^2
estat icc



*-------------------------------------------------------------------------------
* Partial Model 2
*-------------------------------------------------------------------------------

* Fit the model
mepoisson y i.insurance, exposure(n) || inter:, vce(robust) irr

* Store the estimation results
estimates store m2

* Display information criteria
estat ic

* Predict the number of teenage mothers
predict m2n, mu
generate m2ir = m2n/n

* Calculate the ROC
expand 2
bysort inter: generate y01 = _n - 1
generate int n01 = .
```

```
replace n01 = n - y if y01 == 0

replace n01 = y if y01 == 1

roctab y01 m2ir [fweight = n01], summary graph

drop y01 n01

duplicates drop


* Calculate the VPC (via Leckie et al. 2020 method)

predict m2xbhat, xb

replace m2xbhat = m2xbhat - ln(n)

scalar m2sigma2u = _b[var(_cons[inter]):_cons]

generate m2mu = exp(m2xbhat + m2sigma2u/2)

generate m2vpc = ((m2mu^2) * (exp(m2sigma2u) - 1)) / ((m2mu^2) * (exp(m2sigma2u) - 1) +
m2mu)

summarize m2vpc [fweight = n]


* Calculate the PCV (for the Poisson versions of the model)

display "PCV = " %3.2f 100*(m2sigma2u - m1sigma2u)/m1sigma2u


* Calculate the VPC (via the latent response formulation of melogit)

meqrlogit y i.insurance ||  inter:, binomial(n)

scalar m2logitsigma2u = exp(_b[lns1_1_1:_cons])^2

estat icc


* Calculate the  PCV (for the logistic versions of the model)

display "PCV = " %3.2f 100*(m2logitsigma2u - m1logitsigma2u) / m1logitsigma2u



*------------------------------------------------------------------------------

* Partial Model 3
```

```
*--------------------------------------------------------------------------------

* Load the stratum level data
use "teentoc_clean_inter.dta", clear

* Fit the model
mepoisson y i.livingarea, exposure(n) || inter:, vce(robust) irr

* Store the estimation results
estimates store m3

* Display information criteria
estat ic

* Predict the number of teenage mothers
predict m3n, mu
generate m3ir = m3n/n

* Calculate the ROC
expand 2
bysort inter: generate y01 = _n - 1
generate int n01 = .
replace n01 = n - y if y01 == 0
replace n01 = y if y01 == 1
roctab y01 m3ir [fweight = n01], summary graph
drop y01 n01
duplicates drop
```

```
* Calculate the VPC (via Leckie et al. 2020 method)

predict m3xbhat, xb

replace m3xbhat = m3xbhat - ln(n)

scalar m3sigma2u = _b[var(_cons[inter]):_cons]

generate m3mu = exp(m3xbhat + m3sigma2u/2)

generate m3vpc = ((m3mu^2) * (exp(m3sigma2u) - 1)) / ((m3mu^2) * (exp(m3sigma2u) - 1) + m3mu)

summarize m3vpc [fweight = n]


* Calculate the PCV (for the Poisson versions of the model)

display "PCV = " %3.2f 100*(m3sigma2u - m1sigma2u)/m1sigma2u


* Calculate the VPC (via the latent response formulation of melogit)

meqrlogit y i.livingarea ||  inter:, binomial(n)

scalar m3logitsigma2u = exp(_b[lns1_1_1:_cons])^2

estat icc


* Calculate the  PCV (for the logistic versions of the model)

display "PCV = " %3.2f 100*(m3logitsigma2u - m1logitsigma2u) / m1logitsigma2u



*------------------------------------------------------------------------------

* Partial Model 4

*------------------------------------------------------------------------------


* Load the stratum level data

use "teentoc_clean_inter.dta", clear


* Fit the model
```

```
mepoisson y i.region, exposure(n) || inter:, vce(robust) irr

* Store the estimation results
estimates store m4

* Display information criteria
estat ic

* Predict the number of teenage mothers
predict m4n, mu
generate m4ir = m4n/n

* Calculate the ROC
expand 2
bysort inter: generate y01 = _n - 1
generate int n01 = .
replace n01 = n - y if y01 == 0
replace n01 = y if y01 == 1
roctab y01 m4ir [fweight = n01], summary graph
duplicates drop
drop y01 n01

* Calculate the VPC (via Leckie et al. 2020 method)
predict m4xbhat, xb
replace m4xbhat = m4xbhat - ln(n)
scalar m4sigma2u = _b[var(_cons[inter]):_cons]
generate m4mu = exp(m4xbhat + m4sigma2u/2)
generate m4vpc = ((m4mu^2) * (exp(m4sigma2u) - 1)) / ((m4mu^2) * (exp(m4sigma2u) - 1) +
m4mu)
```

```
summarize m4vpc [fweight = n]

* Calculate the PCV (for the Poisson versions of the model)
display "PCV = " %3.2f 100*(m4sigma2u - m1sigma2u)/m1sigma2u

* Calculate the VPC (via the latent response formulation of melogit)
meqrlogit y i.region ||  inter:, binomial(n)
scalar m4logitsigma2u = exp(_b[lns1_1_1:_cons])^2
estat icc

* Calculate the  PCV (for the logistic versions of the model)
display "PCV = " %3.2f 100*(m4logitsigma2u - m1logitsigma2u) / m1logitsigma2u


*------------------------------------------------------------------------------
* Partial Model 5
*------------------------------------------------------------------------------

* Fit the model
mepoisson y i.etnia, exposure(n) || inter:, vce(robust) irr

* Store the estimation results
estimates store m5

* Display information criteria
estat ic

* Predict the number of teenage mothers
predict m5n, mu
```

```
generate m5ir = m5n/n

* Calculate the ROC
expand 2
bysort inter: generate y01 = _n - 1
generate int n01 = .
replace n01 = n - y if y01 == 0
replace n01 = y if y01 == 1
roctab y01 m5ir [fweight = n01], summary graph
drop y01 n01
duplicates drop

* Calculate the VPC (via Leckie et al. 2020 method)
predict m5xbhat, xb
replace m5xbhat = m5xbhat - ln(n)
scalar m5sigma2u = _b[var(_cons[inter]):_cons]
generate m5mu = exp(m5xbhat + m5sigma2u/2)
generate m5vpc = ((m5mu^2) * (exp(m5sigma2u) - 1)) / ((m5mu^2) * (exp(m5sigma2u) - 1) +
m5mu)
summarize m5vpc [fweight = n]

* Calculate the PCV (for the Poisson versions of the model)
display "PCV = " %3.2f 100*(m5sigma2u - m1sigma2u)/m1sigma2u

* Calculate the VPC (via the latent response formulation of melogit)
meqrlogit y i.etnia || inter:, binomial(n)
scalar m5logitsigma2u = exp(_b[lns1_1_1:_cons])^2
estat icc
```

```
* Calculate the  PCV (for the logistic versions of the model)
display "PCV = " %3.2f 100*(m5logitsigma2u - m1logitsigma2u) / m1logitsigma2u



*-----------------------------------------------------------------------------
* Intersectional Full Model 6
*-----------------------------------------------------------------------------


* Load the stratum level data
use "teentoc_clean_inter.dta", clear


* Fit the model
mepoisson y i.insurance i.livingarea i.region i.etnia, exposure(n) || inter:, vce(robust) irr


* Store the estimation results
estimates store m6


* Display information criteria
estat ic


* Predict the number of teenage mothers
predict m6n, mu
generate m6ir = m6n/n


* Calculate the ROC
expand 2
bysort inter: generate y01 = _n - 1
assert inlist(y01, 0, 1)
generate int n01 = .
```

```
replace n01 = n - y if y01 == 0

replace n01 = y if y01 == 1

roctab y01 m6ir [fweight = n01], summary graph

drop y01 n01

duplicates drop


* Calculate the VPC (via Leckie et al. 2020 method)

predict m6xbhat, xb

replace m6xbhat = m6xbhat - ln(n)

scalar m6sigma2u = _b[var(_cons[inter]):_cons]

generate m6mu = exp(m6xbhat + m6sigma2u/2)

generate m6vpc = ((m6mu^2) * (exp(m6sigma2u) - 1)) / ((m6mu^2) * (exp(m6sigma2u) - 1) +
m6mu)

summarize m6vpc [fweight = n]


* Calculate the PCV (for the Poisson versions of the model)

display "PCV = " %3.2f 100*(m6sigma2u - m1sigma2u)/m1sigma2u


* Calculate the VPC (via the latent response formulation of melogit)

meqrlogit y i.insurance i.livingarea i.region i.etnia|| inter:, binomial(n)

scalar m6logitsigma2u = exp(_b[lns1_1_1:_cons])^2

estat icc


* Calculate the  PCV (for the logistic versions of the model)

display "PCV = " %3.2f 100*(m6logitsigma2u - m1logitsigma2u) / m1logitsigma2u



*------------------------------------------------------------------------------

* Figure of interaction
```

```
*-------------------------------------------------------------------------------

use "teentoc_clean_inter.dta", clear
mepoisson y i.insurance i.livingarea i.region i.etnia, exposure(n) || inter:, vce(robust) irr
predict m6u, reffects reses(m6use)
keep inter m6u m6use
duplicates drop
generate m6lo = m6u - 1.96*m6use
generate m6hi = m6u + 1.96*m6use

twoway ///
	(scatter m6u inter, mcolor(black)) ///
	(pcspike m6hi inter m6lo inter, lcolor(black)) ///
	, ///
	ytitle("Predicted random effect") ///
	yline(0) ///
	ylabel(-.6(.2).6, angle(horizontal) format(%2.1f)) ///
	xlabel(1(1)60, labsize(vsmall) angle(ninety)) ///
	legend(off) ///
	scheme(s1mono)

* Export graph
graph save "Figure5.gph", replace
graph export "Figure5.png", replace width(1000)



********************************************************************************
* (2) MAIHDA Geographical
```

```
*******************************************************************************

*------------------------------------------------------------------------------
* Null Model 7
*------------------------------------------------------------------------------

* Load the department level data
use "teentoc_clean_departo.dta", clear

* Fit the model
mepoisson y, exposure(n) || departo:, vce(robust) irr

* Store the estimation results
estimates store m7

* Display information criteria
estat ic

* Predict the number of teenage mothers
predict m7n, mu
generate m7ir = m7n/n

* Calculate the ROC
expand 2
bysort departo: generate y01 = _n - 1
assert inlist(y01, 0, 1)
generate int n01 = .
replace n01 = n - y if y01 == 0
replace n01 = y if y01 == 1
```

```
roctab y01 m7ir [fweight = n01], summary graph

drop y01 n01

duplicates drop


* Calculate the VPC (via Leckie et al. 2020 method)

predict m7xbhat, xb

replace m7xbhat = m7xbhat - ln(n)

scalar m7sigma2u = _b[var(_cons[departo]):_cons]

generate m7mu = exp(m7xbhat + m7sigma2u/2)

generate m7vpc = ((m7mu^2) * (exp(m7sigma2u) - 1)) / ((m7mu^2) * (exp(m7sigma2u) - 1) +
m7mu)

summarize m7vpc [fweight = n]


* VPC: Latent response formulation of mixed-effects logistic regression

meqrlogit y || departo:, binomial(n)

scalar m7logitsigma2u = exp(_b[lns1_1_1:_cons])^2

estat icc



*******************************************************************************

exit
```
